# Connecting in Crisis: Enhancing Family Satisfaction in the Intensive Care Unit Through Effective Communication in an Indian Tertiary Care Hospital

**DOI:** 10.7759/cureus.82683

**Published:** 2025-04-21

**Authors:** Mehul Shah, Kishor Surenderan, Gauri Pathare, Shreyans Rai, Mayur Patel, Darshana Rathod, Rahul Pandit, Arindham Kar, Tushar Parmar, Hirak Patel

**Affiliations:** 1 Department of Critical Care Medicine, Sir H. N. Reliance Foundation Hospital and Research Centre, Mumbai, IND; 2 Sir H. N. Medical Research Society, Sir H. N. Reliance Foundation Hospital and Research Centre, Mumbai, IND; 3 Department of Academics and Research, Sir H. N. Reliance Foundation Hospital and Research Centre, Mumbai, IND; 4 Department of Medical Services, Sir H. N. Reliance Foundation Hospital and Research Centre, Mumbai, IND

**Keywords:** critically ill, family satisfaction, intensive care unit, quality assessment in healthcare, quality improvement, quality of care

## Abstract

Background

Patient satisfaction is an important indicator of the quality of healthcare. However, in critically ill patients who are unable to actively participate in the decision-making process or provide feedback, the satisfaction of their family members is important. The modified Family Satisfaction in the Intensive Care Unit (FS-ICU-24R) questionnaire is a globally validated tool to measure the quality of care in the intensive care unit (ICU) from the perspective of the patient’s family. Therefore, this study aimed to evaluate the performance of our tertiary care center using this questionnaire and identify factors influencing family satisfaction in the ICU and areas requiring improvement to improve healthcare quality.

Methodology

This retrospective cohort study was conducted in the ICU of an Indian tertiary care hospital. Data were collected from the FS-ICU-24R surveys administered to adult family members of critically ill patients admitted to the ICU for at least seven days between January 1, 2019, and October 31, 2024. Family satisfaction was further subdivided into satisfaction with care (FS-Care) and decision-making (FS-DM) domains based on the FS-ICU-24R questionnaire. To enhance family satisfaction, our hospital implemented two quality improvement initiatives in 2022: (1) communication skills workshops for ICU staff and (2) multidisciplinary meetings with the families of long-stay ICU patients. Descriptive statistics were used to characterize participant and patient characteristics and family satisfaction scores. Differences between satisfaction levels were analyzed using a two-sample t-test. Spearman’s rho was used to assess the correlation of patient and participant characteristics with family satisfaction.

Results

This study included 614 participants with a 98.5% (605/614) response rate. We found a high overall family satisfaction level (86.83 ± 19.52), with higher satisfaction with FS-Care (87.7 ± 19.0) than FS-DM (85.4 ± 20.4). A similar trend was observed when the patient cohort was grouped based on the need for mechanical ventilation. While we found no significant difference in the family satisfaction levels based on ventilation status, families of non-ventilated patients were more satisfied with the consideration provided by the ICU staff and the communication frequency with nursing staff than the families of ventilated patients. Family satisfaction levels did not correlate with any participant or patient characteristic. Considering our tertiary care status, a majority (~31%, 154/502) of the patient cohort had high APACHE II scores reflecting their critical condition, with 78% (455/582) requiring mechanical ventilation. Implementation of quality improvement measures resulted in significant improvements in both FS-Care (84.8 ± 18.6 vs. 88.3 ± 19.0) and FS-DM (79.7 ± 22.4 vs. 86.5 ± 19.8) domains.

Conclusions

While the family satisfaction of patients admitted to the ICU in our hospital was relatively high, there remain areas for improvement. The satisfaction level with the FS-DM domain was lower compared to that for the FS-Care domain; however, this trend has been observed in healthcare institutions globally. Moreover, as our hospital is a tertiary institute, the patient population comprises more critical cases that require immediate treatment, reducing the time that can be afforded to make decisions. Nevertheless, the implementation of quality improvement measures enhanced the family satisfaction levels.

## Introduction

Patient satisfaction is the most important indicator of the quality of healthcare [1]. It is a key measure for assessing the performance of healthcare professionals and the quality of healthcare delivered at an organization. However, patients admitted to the intensive care unit (ICU) are critically ill and, in most cases, are unable to actively participate in the decision-making process or provide feedback. In such cases, the patient’s family is the main point of contact, and their opinions and experiences should be considered. The family’s perspective is central to assessing the level of medical care provided in the ICU. Thus, family satisfaction is a key measure of healthcare quality in the ICU.

Recent years have seen a rise in public awareness regarding the varying degree of quality among healthcare providers. In particular, people value empathetic communication and human connection in healthcare settings, along with transparency in terms of quality of care, provider performance, and outcomes. This has prompted healthcare organizations to continually assess and improve their services with the goal of providing high-quality healthcare services. Surveys are valuable tools to evaluate consumer satisfaction and can be implemented in healthcare services to evaluate patient satisfaction, understand their expectations, receive suggestions and feedback, and thereby improve the quality of healthcare services. Indeed, several tools have been developed to assess family satisfaction [2]. However, the most widely validated is the Family Satisfaction in the Intensive Care Unit (FS-ICU) questionnaire, which was subsequently refined and modified in 2018 (FS-ICU-24R) [3]. The FS-ICU-24R questionnaire includes self-rated levels of satisfaction on overall ICU experience, communication, and decision-making, and it was found to demonstrate good psychometric properties [4]. Another study from the United Kingdom found that the FS-ICU-24 questionnaire appropriately represents the topics and themes identified as important by the family members of ICU patients and is, therefore, suitable as a tool to measure family satisfaction with the ICU [5].

Studies on family satisfaction in the critical care setting are limited, especially in the Indian setting. Indian families differ significantly from their global counterparts culturally as well as socially, and their expectations, needs, and stressors associated with hospitalization are likely to be different. However, studies exploring the perception of family members of ICU patients in India are scarce. The present study aimed to evaluate the family satisfaction of patients admitted to the ICU of a tertiary care center in India for more than seven days, to improve our understanding and thereby facilitate a better experience for the families of critically ill patients.

## Materials and methods

Study design

This is a retrospective cohort study analyzing the FS-ICU-24R surveys conducted in the ICU of Sir H. N. Reliance Foundation Hospital and Research Centre, Mumbai, India, between January 1, 2019, and October 31, 2024.

Study population

Adult family members (aged >18 years) of patients admitted to the ICU for ≥7 days were included in this study. The FS-ICU-24R questionnaires [6] were given to a family member during their visit to the ICU. When multiple relatives were present, the questionnaire was administered to the individual who was identified as the primary contact or who had visited the patient most frequently. The study protocol was reviewed and approved by the Institutional Ethics Committee of Sir H. N. Reliance Foundation Hospital and Research Centre (approval number: HNH/IEC/2024/OCS/CCM/153), and the requirement of informed consent was waived considering that the survey is a quality assessment tool and does not disclose patient or participant information. Patient characteristics, including demographic details and clinical characteristics, such as ventilation status, ICU admission type, and Acute Physiology and Chronic Health Evaluation II (APACHE II) score, were obtained from the electronic surveillance system of the hospital. Participant demographics were obtained from the survey questionnaire.

Data collection

The results of the FS-ICU-24R survey were compiled in a secure web-based application, Research Electronic Data Capture (REDCap) (https://ceru.hpcvl.queensu.ca/EDC/redcap_survey/redcap_v8.5.16/DataExport/index.php?pid=23&report_id=10&stats_charts=1), which was developed by Vanderbilt University to capture data for clinical research and create databases and projects [7]. As all participants in the study were proficient in English, no linguistic adaptation of the FS-ICU-24R was required.

Quality improvement measures

To improve family satisfaction, our hospital has introduced two initiatives since 2022. First, the hospital conducted a workshop series on communication skills improvement in 2022 for the ICU staff. Second, for all critically ill patients who exceed seven days of ICU stay, a multi-disciplinary meeting with the patient’s family is conducted to discuss the care plan. The multi-disciplinary team includes all specialty doctors, including surgeons, involved with patient care, nursing staff, as well as the finance department. Further, family members are updated about the daily care plan, which is countersigned by the family member to ensure query resolution daily.

Data analysis

Data were systematized as previously described for rescoring of the FS-ICU-24 questionnaire [8]. Accordingly, the Likert scale 1-5 was recoded to a new scale with scores 0-100, where 0 = poor, 25 = fair, 50 = good, 75 = very good, and 100 = excellent. Descriptive statistics (frequency and percentage or mean ± standard deviation) were used to characterize participant and patient characteristics as well as Family Satisfaction (FS) scores. Categorical variables were compared using the chi-square test. Differences between FS levels based on any parameter were analyzed using a two-sample t-test. Differences between groups were assessed using one-way analysis of variance (ANOVA). Spearman’s rho was used to determine the correlation coefficient between all the items in the questionnaire and overall satisfaction. All analyses were performed using the SPSS statistical software (version 29.0, IBM Corp., NY, USA).

## Results

Participant and patient demographics

A total of 605 participants were included in this study. The response rate for the survey was 98.5% (605/614). A majority (71.3%) of the participants were between 31 and 60 years of age, while 16.4% and 12.3% were aged <30 and >60 years, respectively. The proportion of male (54.7%) and female (45.3%) respondents did not significantly differ. Most (90.6%) of the participants were direct relatives of the patients; of the respondents, 51.4% were offspring, 26.1% were spouses, 4.4% were parents, and 8.7% were siblings, while 8.7% were secondary relatives. As some questions were left unanswered by the participants, the total frequency of responses for each item in the questionnaire differed. Most of the participants were educated, with 43.6% and 32.6% holding a university and graduate degree, respectively; 11.3% and 8.1% with basic schooling and technical/diploma education, respectively; while only 4.5% of the participants did not complete secondary education.

Table 1 shows the demographic characteristics of the patients admitted to the ICU. Our patient cohort predominantly comprised males (65%) over the age of 60 years (57.1%). Over 60% of the ICU population underwent elective surgeries, while only 4% required emergency surgeries. Based on APACHE II score, the majority of our patient cohort was at a moderate (48%) or high (30%) risk of mortality, which is consistent with the fact that we receive sicker, critically ill patients, considering that our hospital is a tertiary center. Further, 85.3% of the patient cohort required mechanical ventilation.

**Table 1:** Patient characteristics. APACHE II score: 0–10: low risk (patients in this range have a relatively low risk of mortality); 11–20: moderate risk (patients in this range have a moderate risk of mortality); 21–30: high risk (patients in this range have a high risk of mortality); ≥31: very high risk (patients in this range have a very high risk of mortality). APACHE II: Acute Physiology and Chronic Health Evaluation II

Patient characteristic	Frequency (%)
Age	
≤30 years	23 (3.96%)
31–60 years	226 (38.89%)
>60 years	332 (57.14%)
Sex (male/female)	377 (65.0%) / 203 (35.0%)
Type of admission	
Medical	187 (32.2%)
Surgical elective	370 (63.7%)
Surgical emergency	24 (4.1%)
Presence of comorbidities	
Yes	488 (85.3%)
No	84 (14.7%)
Mechanical ventilation	
Yes	455 (78.2%)
No	127 (21.8%)
APACHE II score	
1–10	107 (21.3%)
11–20	241 (48.0%)
21–30	150 (29.9%)
31–40	4 (0.8%)

Family satisfaction levels

**Figure 1:**
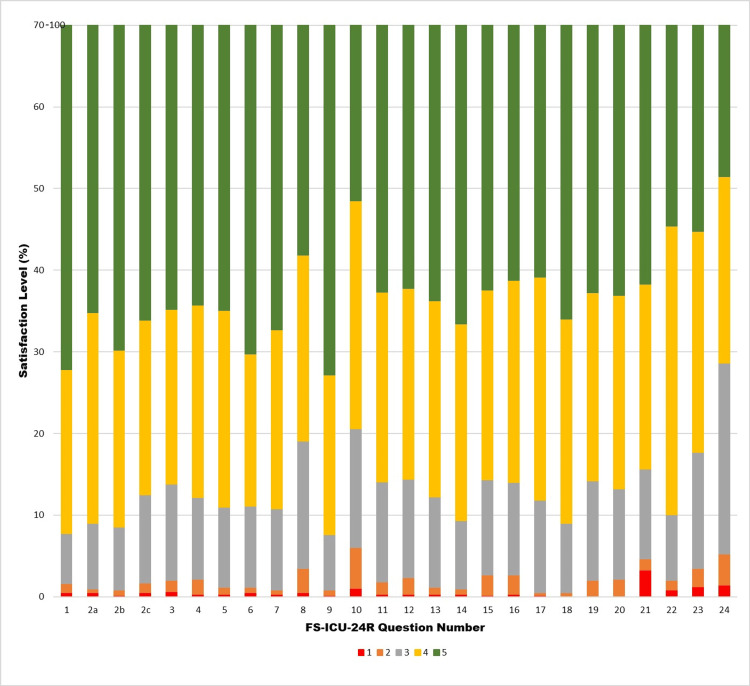
Family satisfaction with ICU care: responses to FS-ICU-24R survey questions. The distribution of participant responses to the survey questions with respect to the five levels of satisfaction is shown. Satisfaction levels: 1, very dissatisfied; 2, slightly dissatisfied; 3, mostly satisfied; 4, very satisfied; 5, completely satisfied. ICU: intensive care unit; FS-ICU-24R: Family Satisfaction in the Intensive Care Unit

The FS-ICU-24R can be subdivided into satisfaction with care (FS-Care) and satisfaction with the decision-making process (FS-DM). In our cohort, we observed overall satisfaction with care provided in the ICU (Figure 1). We found that 98.2% of the participants reported satisfaction with the FS-Care domain, while 97.4% reported satisfaction with the FS-DM domain. To further analyze the satisfaction levels, the Likert scale used in the FS-ICU-24R questionnaire was recoded to a 100-point scale (Table 2). The highest satisfaction was observed for care and support provided by the ICU staff, as well as their competence and communication frequency. The main areas that showed dissatisfaction were related to the decision-making process (Q21-Q24).

**Table 2:** Family satisfaction with ICU care. ^a^: Responses to the FS-ICU-24R questionnaire recorded using Likert scale 1–5 were recoded to a new scale with score 0–100, where 0 = poor, 25 = fair, 50 = good, 75 = very good, and 100 = excellent. ICU: intensive care unit; FS-ICU-24R: Family Satisfaction in the Intensive Care Unit

Question/Item		Mean (SD) (rescaled value^a^)
FS-Care		87.7 (19.0)
Q1	Concern and caring by ICU staff: the courtesy, respect, and compassion your family member (the patient) was given	90.66 (17.53)
Q2A	How well the ICU staff assessed and treated your family member’s pain?	88.87 (17.45)
Q2B	How well the ICU staff assessed and treated your family member’s breathlessness?	90.04 (16.88)
Q2C	How well the ICU staff assessed and treated your family member’s agitation?	87.54 (19.53)
Q3	Consideration of your needs: how well the ICU staff showed an interest in your needs?	87.16 (20.05)
Q4	Emotional support: how well the ICU staff provided emotional support to you?	87.33 (19.46)
Q5	Co-ordination of care: the teamwork of all the ICU staff who took care of your family member	88.3 (18.25)
Q6	Concern and caring by the ICU staff: the courtesy, respect, and compassion you were given	89.45 (18.38)
Q7	Skill and competence of ICU nurses: how well the nurses cared for your family member?	88.93 (17.96)
Q8	Frequency of communication with ICU nurses: how often nurses communicated to you about your family member’s condition?	83.89 (22.0)
Q9	Skill and competence of ICU doctors (all doctors, including residents): how well doctors cared for your family member?	91.11 (16.35)
Q10	How satisfied are you with the atmosphere (mood) in the ICU waiting room?	80.68 (23.76)
Q11	How satisfied are you with the atmosphere (mood) of the ICU?	86.74 (19.69)
Q12	How satisfied are you with your participation in daily rounds?	86.3 (20.1)
Q13	How satisfied are you with your participation in the care of your critically ill family member?	87.56 (18.7)
Q14	Some people want everything done for their health problems, while others do not want a lot done. How satisfied were you with the level or amount of healthcare your family member received in the ICU?	89.0 (17.56)
FS-DM		85.4 (20.4)
Q15	Frequency of communication with ICU doctors: how often doctors communicated to you about your family member’s condition?	86.37 (20.13)
Q16	Ease of getting information: willingness of the ICU staff to answer your questions.	86.18 (20.16)
Q17	Understanding of information: how well the ICU staff provided you with explanations that you understood?	87.21 (17.9)
Q18	Honesty of information: the honesty of information provided to you about your family member's condition.	89.18 (16.66)
Q19	Completeness of information: how well the ICU staff informed you what was happening to your family member and why things were being done?	86.76 (19.52)
Q20	Consistency of information: the consistency of information provided to you about your family member’s condition (Did you get a similar story from the doctor, nurse, etc.?)	86.99 (19.32)
Q21	How included or excluded did you feel in the decision-making process?	84.5 (24.17)
Q22	How supported did you feel during the decision-making process?	85.42 (19.04)
Q23	Did you feel you had control over the care of your family member?	83.2 (22.18)
Q24	When making decisions, did you have adequate time to have your concerns addressed and questions answered?	78.33 (24.8)
FS-Total		86.83 (19.52)

Effect of quality improvement measures on satisfaction levels

Considering that the FS-ICU-24R questionnaire is a quality improvement tool, we evaluated the feedback from the patient families and implemented measures to address the main issues in 2022. Table 3 shows a comparison of satisfaction levels with FS-ICU-24R questions before and after the implementation of improvement measures by our hospital. First, after conducting the communication skills workshop series for the ICU staff, a significant improvement in satisfaction with emotional support, concern, caring, and consideration toward the family members was observed. Moreover, satisfaction with communication frequency and participation in care increased significantly. Second, the hospital introduced and reinforced “multidisciplinary meetings” with families of long-stay critically ill patients. As a result, the family members were better equipped to make informed decisions after considering all aspects of critical care, including financial burden. This was reflected in a significant increase in family satisfaction levels after 2022 in the FS-DM domain.

**Table 3:** Comparison of family satisfaction before and after implementing improvement measures. Statistical significance was assessed using a two-sample t-test. P-values <0.05 were considered significant. ICU: intensive care unit; FS-ICU-24R: Family Satisfaction in the Intensive Care Unit

Question/Item		Before	After		
		Mean ± SD	Mean ± SD	P-value	t-statistic value
FS-Care		84.7 ± 11.9	87.9± 15.4	0.060	-1.90
Q1	Concern and caring by the ICU staff: the courtesy, respect and compassion your family member (the patient) was given	87.0 ± 21.5	91.0 ± 17.0	0.043	-2.03
2a	How well the ICU staff assessed and treated your family member’s pain?	85.7 ± 16.7	89.2 ± 17.8	0.085	-1.72
2b	How well the ICU staff assessed and treated your family member’s breathlessness?	89.0 ± 15.5	89.96 ± 17.3	0.641	-0.47
2c	How well the ICU staff assessed and treated your family member’s agitation?	82.3± 21.3	88.3 ± 19.1	0.002	-2.63
Q3	Consideration of your needs: how well the ICU staff showed an interest in your needs?	83.2 ± 20.9	87.80 ± 19.9	0.043	-2.02
Q4	Emotional support: how well the ICU staff provided emotional support to you?	82.7 ± 18.1	88.1 ± 19.6	0.016	-2.43
Q5	Coordination of care: the teamwork of all the ICU staff who took care of your family member	87.5 ± 16.3	88.2 ± 18.7	0.737	-0.34
Q6	Concern and caring by the ICU staff: the courtesy, respect and compassion you were given	87.2 ± 17.5	89.7 ± 18.6	0.230	-1.20
Q7	Skill and competence of ICU nurses: how well the nurses cared for your family member?	88.3 ± 16.3	88.9 ± 18.3	0.764	-0.30
Q8	Frequency of communication with ICU nurses: how often nurses communicated to you about your family member’s condition?	77.4 ± 23.3	84.9 ± 21.7	0.003	-3.02
Q9	Skill and competence of ICU doctors (all doctors, including residents): how well doctors cared for your family member?	91.2 ± 15.0	91.0 ± 16.6	0.893	0.13
Q10	How satisfied are you with the atmosphere (mood) in the ICU waiting room?	80.2 ± 20.1	80.7 ± 24.4	0.852	-0.19
Q11	How satisfied are you with the atmosphere (mood) of the ICU?	84.8 ± 17.7	86.9 ± 20.1	0.358	-0.92
Q12	How satisfied are you with your participation in daily rounds?	82.3 ± 21.1	86.9 ± 19.9	0.046	-1.99
Q13	How satisfied are you with your participation in the care of your critically ill family member?	83.2 ± 19.1	88.12 ± 18.7	0.021	-2.31
Q14	Some people want everything done for their health problems while others do not want a lot done. How satisfied were you with the level or amount of healthcare your family member received in the ICU?	85.4 ± 17.0	89.6 ± 17.6	0.033	-2.14
FS-DM		79.7 + 14.8	86.2± 15.2	0.000	-3.81
Q15	Frequency of communication with ICU doctors: how often doctors communicated to you about your family member’s condition?	83.5 ± 21.9	86.8 ± 19.8	0.143	-1.46
Q16	Ease of getting information: willingness of the ICU staff to answer your questions.	81.7 ± 21.4	86.9 ± 19.9	0.021	-2.31
Q17	Understanding of information: how well the ICU staff provided you with explanations that you understood?	83.3 ± 18.6	87.8 ± 17.8	0.030	-2.19
Q18	Honesty of information: the honesty of information provided to you about your family member’s condition.	85.5 ± 17.8	89.8 ± 16.4	0.023	-2.30
Q19	Completeness of information: how well the ICU staff informed you what was happening to your family member and why things were being done?	80.7 ± 22.5	87.8 ± 18.8	0.001	-3.23
Q20	Consistency of information: the consistency of information provided to you about your family member’s condition (did you get a similar story from the doctor, nurse, etc.?)	81.3 ± 21.8	87.9 ± 18.8	0.002	-3.03
Q21	How included or excluded did you feel in the decision making process?	77.9 ± 28.2	85.8 ± 23.1	0.004	-2.90
Q22	How supported did you feel during the decision making process?	79.7 ± 21.1	86.5 ± 18.4	0.002	-3.16
Q23	Did you feel you had control over the care of your family member?	76.5 ± 24.5	84.4 ± 21.6	0.002	-3.09
Q24	When making decisions, did you have adequate time to have your concerns addressed and questions answered?	67.2 ± 26.4	80.4 ± 24.0	0.000	-4.71

Family satisfaction based on the ventilation status of the patient

**Figure 2:**
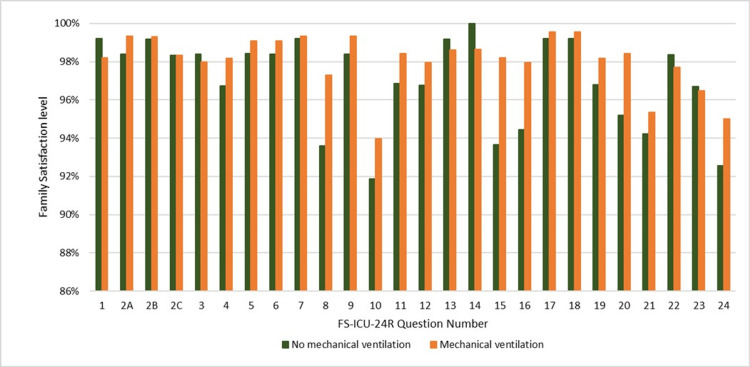
Family satisfaction for items in the FS-ICU-24R questionnaire based on the ventilation status of the patient. ICU: intensive care unit; FS-ICU-24R: Family Satisfaction in the Intensive Care Unit

We compared the family satisfaction levels based on the requirement of mechanical ventilation for the ICU patients (Figure 2). We found no significant difference between the family satisfaction levels of ventilated (FS-Care: 98.3%, 86.0 ± 19.8; FS-DM: 97.7%, 83.5 ± 22.1) and non-ventilated (FS-Care: 97.7%, 88.3 ± 18.8; FS-DM: 96.0%, 85.9 ± 20.0) patients. However, families of non-ventilated patients were more satisfied with the consideration and support provided to them, the extent of their participation in the patient’s care, and care provided to the patient than the families of ventilated patients. Families of ventilated patients were more satisfied with how the ICU staff responded to their need for information regarding the patient, their level of participation in the daily rounds, as well as the time provided for decision-making, than the families of non-ventilated patients.

As ventilated patients comprised 78.2% of our total patient cohort, we separately assessed the family satisfaction in this group. While the overall satisfaction levels remained high, the main areas where families of ventilated patients were dissatisfied were the frequency of communication with the ICU staff and the level of their participation in daily rounds in the FS-Care domain; in the FD-DM domain, they were dissatisfied with the time provided to make decisions, the level of inclusion in the decision-making process, and the level of control over patient care. The family members of ventilated patients were most satisfied with how well the ICU staff explained the patient-related information, as well as the honesty of the information regarding the patient’s condition. A comparison of family satisfaction before and after implementing improvement measures by our hospital revealed that after 2022, satisfaction level with the decision-making process increased significantly (FS-DM: 95.6% vs 97.9%, p < 0.001, two-sample t-test).

For non-ventilated patients, the areas of family dissatisfaction with care (FS-Care) included frequency of communication with ICU nurses and emotional support provided by the ICU staff; concerning decision-making (FS-DM), they were dissatisfied with the time provided to make decisions, communication frequency with doctors, inclusion in the decision-making process, and ease of obtaining information from the ICU staff as well as the consistency of information. Notably, the family members were the most satisfied with the level of medical care provided to the patient.

Family satisfaction based on the type of ICU admission

When compared in terms of the type of ICU admission, there was no significant difference in the family satisfaction levels for surgical and non-surgical patients (Table 4). As expected, satisfaction with decision-making was higher for patients who underwent elective surgeries compared to those who underwent emergency surgeries. The FS-DM levels for non-surgical patients and those who underwent elective surgeries were comparable. Interestingly, family members of patients who underwent elective surgery were less satisfied with the care and concern toward the patient (84.4 ± 17.8, p < 0.05), as well as the assessment and treatment of patient’s breathlessness (83.3 ± 19.0, p < 0.05) than the families of non-surgical (91.0 ± 17.5; 92.1 ± 15.1) and surgical patients (91.4 ± 16.9; 89.7 ± 17.4).

**Table 4:** Comparison of satisfaction levels (FS-Care and FS-DM) based on the type of ICU admission. The difference between the groups was assessed using analysis of variance. P-values <0.05 were considered statistically significant. ICU: intensive care unit; FS-ICU-24R: Family Satisfaction in the Intensive Care Unit

Type of ICU admission	Non-surgical (medical)	Surgical emergency	Surgical elective		
	Mean (SD)	Mean (SD)	Mean (SD)	P-value	F-statistic value
FS-Care	88.26 (18.2)	87.81 (19.31)	83.03 (19.85)	0.290	1.24
FS-DM	85.43 (20.84)	85.63 (20.21)	81.77 (22.71)	0.505	0.68

## Discussion

There is increasing consensus regarding family satisfaction with ICU care as an accepted measure of quality of care [9], and FS-ICU ratings have been validated in several countries. The present study reports family satisfaction with ICU care in a tertiary care center in western India. While there have been previous studies on family satisfaction from India [10,11], none have focused on the ventilation status of the patients or the extended duration of ICU stay. We found high overall satisfaction with the care provided in the ICU. However, the satisfaction level was higher for the FS-Care subscale than for FD-DM. The main areas in which the participants were dissatisfied were related to the decision-making process. In particular, a majority of the dissatisfied cohort felt excluded from the decision-making process and found the time provided to reach a decision to be inadequate. Notably, the participants were satisfied with the information provided to facilitate the decision-making process. In our cohort, we did not find any significant correlation between the family satisfaction levels and any participant or patient characteristic.

International consensus on the benefits of patient and family engagement in the ICU is gaining popularity. Notably, the overall family satisfaction level in our study cohort (97.9%; 86.8 ± 19.5) is higher than that reported in Italian (84.8 ± 11.6) [12], Australian (96.8%) [13], Korean (75.4   ± 17.7) [14], Norwegian (74.1 ± 15.2) [15], Canadian [8], and United Kingdom (79.7 ± 16.7) [16] cohorts. The satisfaction levels in our cohort were higher than those reported previously from India [10,11]. However, the cohort selected by Bharadwaj et al. comprised families of patients with neurological illness, and their lower satisfaction rates may be due to the highly morbid nature of illness in their kin [10]. The level of family satisfaction with care was higher than that with the decision-making process in the present study, which was consistent with the findings of Canadian [8], Australian [13], and Norwegian [15] studies. However, a multicenter study from South Korea reported greater satisfaction with information/decision-making than with care [14]. In our cohort, the level of education of the participants did not influence the satisfaction levels. In contrast, in a multi-center, cross-sectional study from Ethiopia, education levels were found to be associated with family satisfaction [17]. Nevertheless, despite the high literacy rate of our study cohort, a lack of medical knowledge and understanding seemed to result in dissatisfaction, especially with the decision-making domain.

The FS-IC-24R survey was launched in our hospital in 2019 with the goal of quality improvement. Accordingly, after reviewing the initial feedback, our hospital conducted a workshop series on communication skills in 2022 to improve the communication between the ICU nursing staff and patient families, focusing on clear, concise, correct, complete, consistent, and courteous communication. This initiative yielded successful results, and a marked improvement in the FS-Care domain and increased satisfaction with communication has been observed since 2022. The main areas that showed a significant improvement in family satisfaction were consideration, care, respect, compassion, and emotional support provided by ICU staff, as well as communication frequency with nursing staff. Satisfaction levels were also significantly higher for participant involvement in the care of the patient. Thus, we found that educating the ICU staff regarding the importance of communication led to improved interaction between the ICU staff and patient family, which was reflected in the increase in satisfaction levels.

To improve our performance in the decision-making domain, we implemented “multidisciplinary meetings” between all doctors involved in the treatment of the patient since admission, nursing staff, finance department, and the family of patients who have exceeded seven days of ICU stay. This meeting is set up to explain the patient’s current situation, complications, prognosis, and future course of action, and helps the patient’s family make the necessary decisions regarding the future care plan. The patient’s family is also apprised of the potential costs involved and availability of financial aid. The involvement of the finance department in this meeting is particularly noteworthy as the financial burden of hospitalization is a major source of stress to the patient’s family. A significant increase in family satisfaction was observed in the FS-DM domain since the implantation of the multidisciplinary meeting; however, a marked improvement was observed for satisfaction with inclusion in the decision-making process (Q21), but it was statistically non-significant. A major reason for this may be a lack of medical knowledge and understanding (irrespective of the educational status), which may make the family feel excluded from the decision-making process, despite getting the relevant information regarding the treatment from the ICU staff. Indeed, the family members’ understanding of the clinical problem was predominantly very poor (>65%), especially in the case of patients on ventilator support, dialysis, and prolonged ICU stay, requiring multiple counselling sessions. Another reason is the inherent urgency to treat critically ill patients; in most cases, the treatment window for patients in the ICU is very small, and this is a major factor for family members feeling rushed into making decisions. In fact, ~31% of our patient cohort had a high mortality risk based on the APACHE II score. It is important to note that our institute is a tertiary care center and often admits severely ill patients. Owing to the critical condition of the patients, immediate administration of treatment procedures is inevitable, which, in turn, may afford less time for family members to make decisions. However, we have tried to address this issue with daily communication, which is countersigned by any family member to ensure that they are updated about the daily care plan and that their queries are also resolved. Another issue is the rising trend of seeking a second opinion in India as well as internationally. While second opinions may have certain merits [18], it is not always feasible for critical cases, considering the time constraint for starting treatment. This may lead to dissatisfaction with the time provided for decision-making, as the family members may be unable to obtain a second opinion before making a decision. Thus, counselling family members regarding the clinical aspect of the patient’s condition and conducting multi-disciplinary meetings have resulted in a gradual trend toward improvement in their satisfaction with the FS-DM domain.

The psychological impact of extended hospitalization is profound not only for the patients but also for their family members. Recent years have seen a heightened awareness regarding the adverse psychological outcomes experienced by family members of critically ill patients hospitalized in the ICU [19]. Indeed, Azoulay et al. found that a high risk of post-traumatic stress disorder (PTSD) is common in family members of ICU patients [20]. Moreover, the severity of illness has a negative impact on the mental health of family members [21]. The need for mechanical ventilation is associated with a greater criticality. Therefore, we further evaluated whether the need for mechanical ventilation affects the satisfaction level of patients’ families. Few studies have included ventilation as a criterion, while Hickman et al. included only families of patients requiring mechanical ventilation in their study [22]. To our knowledge, this is the first study to compare the family satisfaction of ventilated and non-ventilated patients in India using the FS-ICU-24R tool. We found no significant difference between the satisfaction levels of ventilated and non-ventilated patients. However, families of non-ventilated patients were more satisfied with the consideration provided by the ICU staff and the communication frequency with the nursing staff than the families of ventilated patients. Interestingly, Naef et al. found that satisfaction with ICU care was strongly associated with family well-being post-ICU [21]. Thus, the ICU staff should note that the family members of ventilated patients are under greater stress and may need to be given more consideration and care. It is noteworthy that after implementing quality improvement measures at our hospital, a marked increase in family satisfaction with the decision-making process was observed in ventilated patients.

We next assessed whether the type of ICU admission has an impact on family satisfaction. There was no difference observed between the family satisfaction levels of medical and surgical patients. However, the family members of patients who had undergone elective surgery were less satisfied with symptom management, in particular breathlessness, of patients in our cohort. This could be attributed to unrealistic expectations for complete resolution of symptoms unrelated to the surgical procedure [23]. As expected, families of patients who underwent elective surgeries had higher satisfaction with decision-making than those who underwent emergency surgeries.

Another strength of the present study is that it evaluated the satisfaction of the family members of patients who were admitted to the ICU for seven or more days. It is interesting to note that in most of the published literature so far, the duration of ICU stay is >48 hours [5,8,10,11,15,24,25]. The length of stay in the ICU is related to the psychological quality of the patients’ family members [26]. Therefore, a longer duration of hospitalization is expected to reflect the true psyche of the patient’s family. By including participants with relatives requiring seven or more days of ICU care, we ensured that the responses to the FS-ICU-24R survey reflected the experiences of families who had a more extended interaction with the ICU staff. The longer duration of interaction likely provided sufficient time for the families to observe and evaluate various aspects of ICU care, leading to more comprehensive and insightful feedback.

A limitation of this study is that the study population did not include families of non-survivors. However, Schwarzkopf et al. found that patient survival was not a predictor of overall satisfaction with the ICU [27]. Khan et al. reached the same conclusion in a six-year retrospective cohort study of family satisfaction with critical care and decision-making in an Australian ICU [13]. Similarly, Salins et al. reviewed 23 articles (systematic review, observational studies, surveys, and qualitative studies) related to family satisfaction with care for non-survivors in the ICU and found that none of these showed ICU survival as a factor influencing family satisfaction [28]. Interestingly, Ferrando et al. [16] and Wall et al. [29] found that families of non-survivors were more satisfied with care in the ICU than families of survivors. However, Haave et al. found patient survival to significantly affect family satisfaction in a cross-sectional study in two Norwegian ICUs; however, the study only included 57 participants [15]. Thus, further investigation into the influence of patient survival on family satisfaction in the ICU is needed.

In summary, while the family satisfaction of patients admitted to the ICU in our hospital was relatively high, there remain areas for improvement. In particular, we observed high satisfaction with the FS-Care domain. The satisfaction level with the FS-DM domain was lower compared to that for the FS-Care domain; however, this trend has been observed in healthcare institutions across the globe. Moreover, our hospital is a quaternary institute and therefore the patient population comprises more critical cases that require immediate treatment, reducing the time that can be afforded to make decisions. Nevertheless, we have ensured the implementation of continual efforts to improve the family satisfaction of ICU patients.

## Conclusions

The hospitalization experience is not limited to the patient-clinician dynamic but also encompasses the experience of family members, especially in the case of critically ill patients. We used FS-ICU-24R as a quality measurement tool to better understand the expectations of the family members of ICU patients and thereby improve our performance. While we have achieved an increase in satisfaction levels after implementing quality improvement measures at our hospital since 2022, it should be noted that quality improvement is an ongoing process. Based on the present study findings, efforts are required to include the family members in the decision-making process related to the critically ill patient. A major strength of our study is that we only included the families of patients requiring extended ICU stay (≥7 days) because the length of ICU stay reflects the degree of criticality of the patient, which, in turn, is directly associated with the mental health of the family members. Another indicator of the severity of illness is the need for mechanical ventilation. While we did not detect a major difference in overall family satisfaction of ventilated and non-ventilated patients, further research is warranted to explore the specific needs of families of ventilated patients. Our study provides insights into the needs and expectations of family members of ICU patients from an Indian perspective. We intend to continue the use of this survey as a tool to highlight the areas of medical care in which we excel, as well as those that need to be improved to continue providing patient/family-centered ICU care. We recommend the implementation of such quality measurement tools, particularly in the Indian healthcare setting, to benchmark performance against global standards and contribute to the standardization of critical care. Moreover, future multicentric studies across diverse healthcare settings with longer-term follow-up and consideration for regional and cultural variations could provide valuable insights into the functioning of the current healthcare system and help identify specific areas that need improvement to enhance the quality of care and family satisfaction in the ICU.
